# Manipulation of TiO_2_ Nanoparticle/Polymer Coatings Wettability and Friction in Different Environments

**DOI:** 10.3390/ma13071702

**Published:** 2020-04-05

**Authors:** Marjetka Conradi, Aleksandra Kocijan, Tadeja Kosec, Bojan Podgornik

**Affiliations:** 1 Institute of Metals and Technology, Lepi pot 11, 1000 Ljubljana, Slovenia; aleksandra.kocijan@imt.si (A.K.); bojan.podgornik@imt.si (B.P.); 2 Slovenian National Building and Civil Engineering Institute, Dimičeva ulica 12, 1000 Ljubljana, Slovenia; tadeja.kosec@zag.si

**Keywords:** polymer coatings, TiO_2_ nanoparticles, wettability, tribology, corrosion

## Abstract

An AISI 316L surface was functionalized by the adsorption of hydrophilic epoxy and epoxy/TiO_2_/epoxy coatings and hydrophobic epoxy/fluoroalkylsilane-functionalized FAS-TiO_2_/epoxy coatings. We characterized the coatings’ wettability, morphology and average surface roughness and discussed the influence of surface wettability and morphology on the coefficient of friction and the wear resistance. Experiments were performed in dry, distilled water and in a simulated physiological solution (Hank’s solution). In the case of dry sliding, a lower coefficient of friction is achieved for both TiO_2_ coatings compared to the pure epoxy coating. In a water environment the same level of friction is shown for all three coatings, whereas in Hank’s solution the friction is reduced for the hydrophilic epoxy/TiO_2_/epoxy coating, increased for the hydrophobic epoxy/FAS-TiO_2_/epoxy coating and has no effect on the pure epoxy coating. The results show that the corrosion resistance is significantly improved for the hydrophobic epoxy/FAS-TiO_2_/epoxy coating compared to the hydrophilic pure epoxy and epoxy/TiO_2_/epoxy coatings.

## 1. Introduction

An efficient way to protect metallic surfaces against adhesive wear and corrosion is the application of various polymer coatings, with epoxy resin being the most commonly used polymer matrix [1,2]. This also makes it possible to manipulate the surface properties with the option to tailor the surface wettability for specific applications to make it hydrophobic (water repellent) or hydrophilic (water attractive) [3,4,5,6,7].

Epoxy coatings, in general, exhibit superior mechanical properties, chemical resistance, good electrical insulation properties and strong adhesion to heterogeneous substrates. Based on a low surface shear resistance and favorable friction characteristics they provide good anti-sticking and anti-galling properties [8]. Furthermore, they serve as an effective physical barrier between the metal and the aggressive environment containing Cl^−^, O_2_ or H^+^ and therefore improve the corrosion resistance and prolong the lifetime of metallic substrates. Pure epoxy coatings are, however, characterized by a highly cross-linked structure, which is responsible for the susceptibility to surface abrasion, scratching and poor resistance to the propagation of cracks in pure epoxy coatings [9,10].

In the past few years, researchers in the field of coatings have, therefore, focused on improving the properties of pure epoxy coatings by implementing various nanofillers [11,12,13,14,15,16]. This included the fabrication of multifunctional coatings with superhydrophobic properties for self-cleaning applications based on the use of various nanoparticles (i.e., nanosilica, TiO_2_, CuO, ZnO). Their properties are tailored via surface multiscale roughness for specific applications [17,18]. Water attractive, superhydrophilic surfaces show the potential for ultrafast drying with special properties, such as antifogging or evaporative cooling [7,19]. Superhydrophilic coatings of TiO_2_ nanoparticles produced by aerosol synthesis also demonstrated self-cleaning and self-disinfecting properties, as reported by De Falco et al. [20]. The photocatalytic properties of TiO_2_ nanoparticles, on the other hand, led to the photoinduced superhydrophilicity of TiO_2_ thin films [21,22] with antimicrobial and antibiofilm efficacy of self-cleaning surfaces [23] or with the possibility for an upgrade to smart coatings, where superhydrophobic and superhydrophilic properties can be manipulated for specific applications through wettability conversion [3].

The modification and ability to control the surface properties, surface roughness and wettability, are, on the other hand, extremely important for various friction applications in different media. Surface properties can be tailored by the application of various inorganic/organic coatings with desirable target properties. However, in general these coatings show low wear resistance. As not a lot of attention has been paid to the combined effect of nanoparticles implementation, roughness and wettability on the surface behaviour of polymer coatings, in this paper we focus on stainless-steel surface functionalization based on the adsorption of polymer coatings with implemented TiO_2_ nanoparticles to study the reinforcement, surface roughness and wettability effect on tribological and corrosion properties of the coated contact surface. A wear analysis of the polymer coatings is performed in dry conditions, distilled water and a physiological Hank’s solution. The coefficient of friction was analysed for a pure epoxy coating in comparison with the sandwich-structured epoxy coatings (epoxy/nanoparticles/epoxy) filled with dual-size, as-received TiO_2_ and fluoroalkylsilane functionalized FAS-TiO_2_ nanoparticles, leading to moderately hydrophilic and hydrophobic surfaces, respectively. The aim of this work was to study the effect of TiO_2_ nanoparticles implementation and functionalization on the tribological and corrosion behaviour of epoxy coatings, not the stainless-steel substrate and therefore detailed analysis of the bare stainless-steel substrate was not carried out.

## 2. Materials and Methods

Materials—Austenitic stainless steel AISI 316L (17% Cr, 10% Ni, 2.1% Mo, 1.4% Mn, 0.38% Si, 0.041% P, 0.021% C, <0.005% S in mass fraction) was used as the substrate. A two-component USP Class-VI biocompatible epoxy EPO-TEK 302-3M (EPOXY TECHNOLOGY, Inc., Billerica, MA, USA) was mixed in the wt.% ratio 100:45. TiO_2_ nanoparticles with mean diameters of 30 nm (anatase) were provided by Cinkarna Celje (Billerica, MA, USA), whereas those with 100 nm (rutile) mean diameter were supplied by US Research Nanomaterials, Inc. (Houston, TX, USA). The nanoparticles were dispersed in ethanol to prepare 3 wt.% TiO_2_ nanoparticle ethanolic solution. The 3 wt.% loading of was chosen to lead to optimized coatings preparation with desirable properties as reported in our previous work [24,25].

Surface modification—TiO_2_ particles were functionalized in a 1% vol ethanolic fluoroalkylsilane (FAS), C_16_H_19_F_17_O_3_Si solution to ensure the hydrophobic effect.

Substrate preparation—1.5 mm-thick steel sheet was cut into discs with a diameter of 25 mm. The steel discs were diamond polished following a standard mechanical procedure and finally ultrasonically cleaned in ethanol.

Coating preparation—Sandwich-like structured coatings, epoxy/TiO_2_/epoxy (shortly TiO_2_ coating) and epoxy/FAS-TiO_2_/epoxy (shortly FAS-TiO_2_ coating), were prepared on a diamond-polished AISI 316L substrate. The first layer of epoxy was spin-coated on the steel surface to ensure the good adhesion of the nanoparticles. In the next step, four double layers of 30 nm and 100 nm TiO_2_/FAS-TiO_2_ nanoparticles were spin-coated on the pre-prepared epoxy layer and finally covered with another thin layer of epoxy for the particle fixation. The coatings were then cured for 3 h at 65 °C. A pure epoxy coating was used as a reference.

Surface characterization—A JEOL JSM-6500F field-emission scanning electron microscope (SEM) with an energy-dispersive X-ray spectrometer (EDS; INCA ENERGY 400, Tokyo, Japan) was used for the morphology evaluation of all the coatings before and after the tribological testing.

The wettability of the coatings was evaluated with static water and Hank’s solution contact-angle measurements. Droplets of water/Hank’s solution with V = 5 µL were deposited on at least three different spots on the coatings to avoid the influence of roughness and gravity on the shape of the droplet. Advex Instruments s.r.o. (Brno, Czech Republic) was used to analyse the droplets and determine the contact angles.

Surface roughness was characterized with an optical 3D metrology system, model Alicona Infinite Focus (Alicona Imaging GmbH, Graz, Austria), and IF-MeasureSuite (Version 5.1) software. At least three measurements on each coating were performed at 20x magnification with a lateral and vertical resolution of 0.9 μm and 50 nm, respectively. The size of the analysed area was 1.337 × 0.993 mm^2^. The average surface roughness, Sa, was evaluated from the general surface-roughness equation:

(1) $$Sa=\frac{1}{L_{x}}\frac{1}{L_{y}}\int_{0}^{L_{x}}\int_{0}^{L_{y}}\left|z\left(x,y\right)\right|dxdy$$

where *L_x_* and *L_y_* are the acquisition lengths of the surface in the *x* and *y* directions and *z*(*x*,*y*) is the height.

Tribological measurements—Tribological testing was performed under a reciprocating sliding motion using a pin-on-disc configuration, where a flat-ended AISI 316L steel pin with a diameter of 5 mm was loaded against a coated stainless-steel plate. Tests were performed at a normal load of 1 N, corresponding to a nominal contact pressure of 20 MPa and an average sliding speed of 5 mm/s. The total sliding distance was up to 1 m, achieved in a sliding time of up to 200 s. Each test was repeated at least three times in order to obtain statistically relevant results. To ensure smooth and parallel contact between the pin and the plate, the pin surface was smoothened before each test using grinding paper (grit 4000) attached to the plate surface and sliding at a load of 12 N for 60 s. After that, the grinding paper was removed and the test continued under the pre-defined contact conditions mentioned above. Regarding the environment, the investigation was focused on three cases. Dry sliding in air in room conditions (T = 20 °C, 50% RH), sliding in distilled water and lastly in the physiological Hank’s solution (the composition is defined below) in order to check applicability of coatings in biomedical applications. In both lubricated cases, the coated steel plate was fully submerged in the lubricant. The test results were evaluated in terms of the sliding time when steady-state friction conditions were reached, the average value of the steady-state coefficient of friction and a wear-scar analysis.

Potentiodynamic measurements—Measurements were performed in a simulated physiological Hank’s solution at pH = 7.8, which contained 8 g/L sodium chloride, 0.40 g/L potassium chloride, 0.35 g/L sodium bicarbonate, 0.25 g/L sodium dihydrogen phosphate dihydrate, 0.06 g/L disodium hydrogen phosphate dihydrate, 0.19 g/L calcium chloride dihydrate, 0.41 g/L magnesium chloride hexahydrate, 0.06 g/L magnesium sulfate heptahydrate and 1 g/L glucose. For measurements a BioLogic Modular Research Grade SP-300 instrument (Seyssinet-Pariset, France) was used. The samples were applied as a working electrode. A saturated calomel electrode (SCE, 0.242 V vs. SHE) was used as the reference electrode and a platinum net as the counter electrode. The samples were stabilized 1 h prior to the measurement at the open-circuit potential (OCP), after which potentiodynamic curves were recorded using a scan rate of 1 mV s^−1^. We repeated all the measurements three times.

## 3. Results and Discussion

### 3.1. Surface Morphology

**Figure 1:**
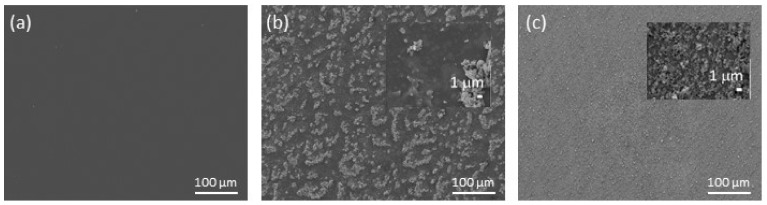
Scanning electron microscope (SEM) images of pure epoxy coating (**a**) and the epoxy/fluoroalkylsilane (FAS)-TiO_2_/epoxy (**b**) and epoxy/TiO_2_/epoxy (**c**) coatings. The insets in (**b**) and (**c**) show the characteristic dimensions of agglomerates.

The morphology differences between all the coatings, i.e., pure epoxy, epoxy/TiO_2_/epoxy and epoxy/FAS-TiO_2_/epoxy, were revealed by SEM imaging (Figure 1). In Figure 1a we can see a smooth, defect-free epoxy coating, while Figure 1b,c show characteristic TiO_2_ nanoparticle agglomerates. In the epoxy/TiO_2_/epoxy coating, the nanoparticles are finely dispersed and agglomerates of the order of sub-micron to micron are observed (Figure 1c). In contrast, in the epoxy/FAS-TiO_2_/epoxy coating with FAS-functionalized TiO_2_ nanoparticles, we observed the formation of large agglomerates up to a few tens of microns (Figure 1b). The morphology differences appear due to the difference in the wetting properties and therefore the incompatibility with the epoxy resin. The as-received TiO_2_ nanoparticles were hydrophilic, which allows a more homogeneous distribution on the hydrophilic epoxy resin. The FAS-TiO_2_ nanoparticles are, on the other hand, hydrophobic due to the –CF_3_ groups and therefore cause the agglomeration of nanoparticles to minimize the surface area on the hydrophilic epoxy resin.

### 3.2. Wetting Properties

Surface wettability was analysed by performing three static contact-angle measurements, θ, with 5 µL droplets of distilled water (θ^W^) and Hank’s solution (θ^Hank^) at different spots on the coatings. As shown in Table 1, the epoxy and TiO_2_ coatings both have a moderately hydrophilic nature in both environments, the water and Hank’s solution, whereas the FAS-TiO_2_ coating is moderately hydrophobic. In addition, the differences in the contact angles between the water/coating and Hank’s solution/coating are not significant, since Hank’s solution is also water based. 

### 3.3. Tribological Behaviour

**Figure 2:**
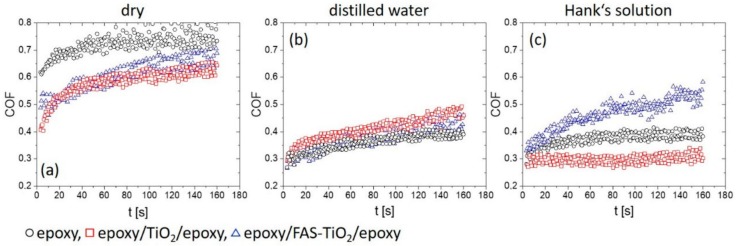
Coefficient of friction under dry sliding. (**a**) Sliding in distilled water (**b**) and in Hank’s solution (**c**) for epoxy, epoxy/FAS-TiO_2_/epoxy, and epoxy/TiO_2_/epoxy coatings.

In the case of the epoxy coating sliding in air, a steady-state coefficient of friction of about 0.73 ± 0.05 was reached after approximately 60 s (Figure 2a), which is in a range of a typical coefficient of friction (0.7–0.8) observed in applications employing uncoated stainless-steel surfaces. When using the coating with TiO_2_ nanoparticles, a steady-state coefficient of friction in air was not achieved before 80 s, but the level of friction was lower than for the epoxy coating, at about 0.62 ± 0.03. A similar level of steady-state friction in air was also shown by the FAS coating (~0.65 ± 0.04), which was achieved after 100 s (Figure 2a).

Shifting to distilled water (Figure 2b) and Hank’s solution (Figure 2c) lubricated contact, coefficient of friction for epoxy coating dropped to 0.40 ± 0.02, with the steady-state conditions reached after about 120 s. No difference between the water and the Hank’s solution could be observed, as shown in Figure 2b,c. However, for the other two coatings reinforced with TiO_2_ nanoparticles the tribological behaviour in the water and the Hank’s solution differs (Figure 2b,c). In the case of TiO_2_ coating sliding in water, steady-state conditions were not reached within 180 s, with friction displaying a steady increase before reaching a slightly higher value as found for pure epoxy coating, of about 0.45 ± 0.03. On the other hand, for the Hank’s solution the steady-state conditions were reached after 20 s with an average value of ~0.30 ± 0.03. The FAS-TiO_2_ coating, being hydrophobic, displays the opposite behaviour in a lubricated contact. In water, a similar steady-state friction of 0.42 ± 0.03 was reached after about 100-120 s, while shifting to Hank’s solution leads to an increase in friction. In this case the steady-state coefficient of friction was above 0.5 ± 0.05, reached after 80 s (Figure 2c).

**Figure 3:**
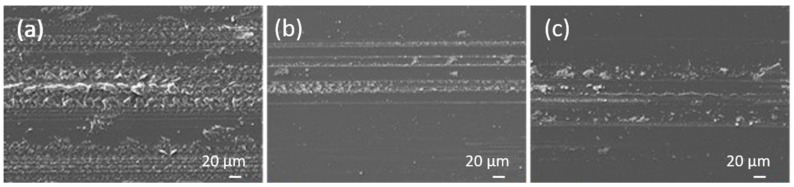
Wear tracks for epoxy coating; (**a**) dry sliding, (**b**) sliding in water and (**c**) sliding in Hank’s solution.

**Figure 4:**
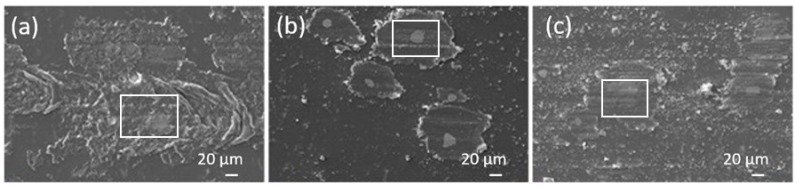
Wear tracks for TiO_2_ coating; (**a**) dry sliding, (**b**) sliding in water and (**c**) sliding in Hank’s solution. Squares mark the energy-dispersive X-ray spectrometer (EDS) measurement sites.

**Figure 5:**
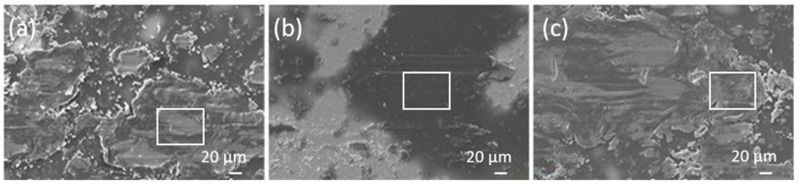
Wear tracks for FAS-TiO_2_ coating; (**a**) dry sliding, (**b**) sliding in distilled water and (**c**) sliding in Hank’s solution. Squares mark the EDS measurement sites.

**Figure 6:**
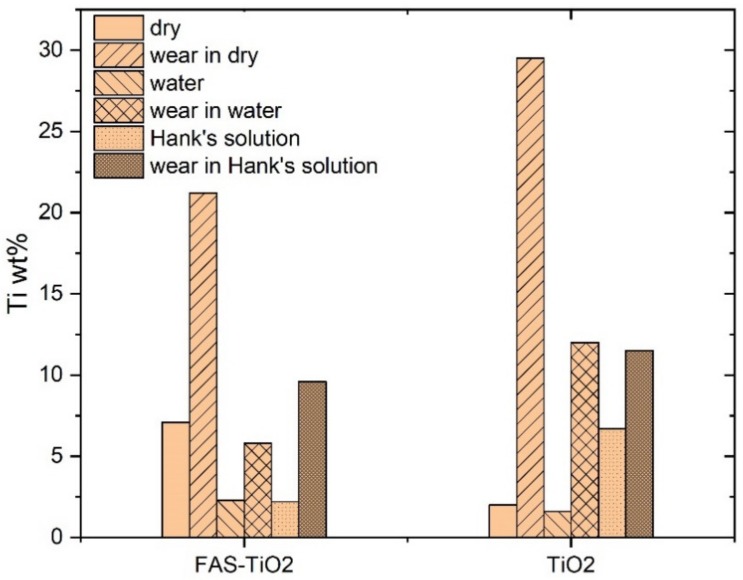
Ti wt.% before and after the tribological experiments in dry conditions, distilled water and Hank’s solution on epoxy/FAS-TiO_2_/epoxy and epoxy/TiO_2_/epoxy coatings, as determined by the EDS analysis. The sites of the EDS measurements for the worn surfaces are marked with squares in Figure 4 and Figure 5.

An analysis of the wear tracks is shown in Figure 3, Figure 4 and Figure 5. In the case of the epoxy coating traces of abrasive wear and the accumulation of wear particles within the wear track can be seen for dry sliding, with the formed transfer films carrying the load, and the abrasive wear mechanism being responsible for the friction level of about 0.7 (Figure 3a). Since the investigation was focused on the polymer coatings’ behaviour, all wear experiments were very short, performed within 5 min, with the wear being concentrated within the coating. In the case of TiO_2_ nanoparticles containing coatings, abrasive wear of the coating followed by coating particles accumulation and transfer film formation is the main wear mechanism. However, in this case coating material starts to accumulate around revealed TiO_2_ nanoparticles forming larger load-carrying low-friction patches (Figure 4a). Similar patches were also observed for the FAS-TiO_2_ coating; however, in this case the patches seem more compact (Figure 5a), taking longer to form and having a higher shear strength and thus resulting in higher friction. The EDS analysis of the patches confirmed an increased concentration of Ti within the patches, which was true for both coatings, as shown in Figure 6.

Shifting to sliding in water, the wear tracks for the epoxy coating look very similar to the dry sliding, except for the substantially reduced abrasive-wear component as well as the coating material transfer-film formation (Figure 3b). The same applies to sliding in the Hank’s solution, as shown in Figure 3c.

When analysing the wear tracks of the TiO_2_ coating sliding in water, patches of the accumulated coating material can still be observed. However, these are much smaller and less frequent (Figure 4b) than found for the dry siding. They are still carrying the load, but the friction level is defined by the water film. In the case of the Hank’s solution, a lot of smaller Ti-containing particles can be observed in addition to the load-carrying patches (Figure 4c), which together with the hydrophilic nature of the coating provide a further reduction in the friction.

Finally, for the FAS-TiO_2_ coating, sliding in water results in a similar wear-track appearance as observed for the TiO_2_ coating, with the number and size of the patches being substantially reduced, compared to the dry sliding (Figure 5b). Again, the patches are carrying the load, but the friction level is defined by the water film, thus resulting in the same friction level for all three coatings (~0.4). However, in the Hank’s solution, large patches start to form again (Figure 5c), partly due to the hydrophobic nature of the coating, thus resulting in an increased friction, in contrast to the TiO_2_ coating.

The results show that TiO_2_ nanoparticles reinforcement provides better polymer coating resistance compared to mechanical and tribological loading which is in agreement with other studied organic coatings with different nanoparticle implementation [26,27]. In our case, improved tribological resistance was provided by the formation of patches of coating material rich in Ti. During initial wear of the epoxy top-layer nanoparticles are exposed, acting as the accelerators of patches formation. As the sliding and wear of the coating progresses coating material starts to accumulate around these initial islands, forming patches of improved load-carrying capacity. At the same time, low-shear strength of the patches results in reduced coefficient of friction under dry sliding in air, although it takes slightly longer before steady-state conditions are reached and the patches of transfer films being formed. Results of lubricated sliding experiments in water and Hank’s solution reveal an important novel contribution showing that, for the epoxy coating, no interactions between the lubricant and the coating take place, thus resulting in almost identical tribological behaviour and friction levels in water and Hank’s solution. This indicates that physical properties of the Hank’s solution (i.e., viscosity, pH, surface tension) are very similar to water and thus have no major influence on the friction behaviour of epoxy-based coatings. The same level of friction is also shown by the TiO_2_ and FAS-TiO_2_ coatings when sliding in water, confirming that regardless of the type of coating and patches formed the friction level in water is defined purely by the water lubrication film (mixed lubrication) without any surface reactions. However, in the Hank’s solution the hydrophilic/hydrophobic nature of the coating together with the reactions between the elements of the coating (revealed TiO_2_ nanoparticles and Ti within the patches) and the solution affect the friction level. For the base epoxy coating, no reactions could be identified, while for the TiO_2_-containing coating with a hydrophilic nature formation of low shear strength hydroxides, i.e., calcium hydroxide [28] have a positive effect on the friction by reducing it by about 20%. However, in the case of functionalized FAS-TiO_2_ coating with a hydrophobic nature more detrimental compounds (which still need to be identified) are formed, increasing friction to over 0.5.

### 3.4. Corrosion Properties

**Figure 7:**
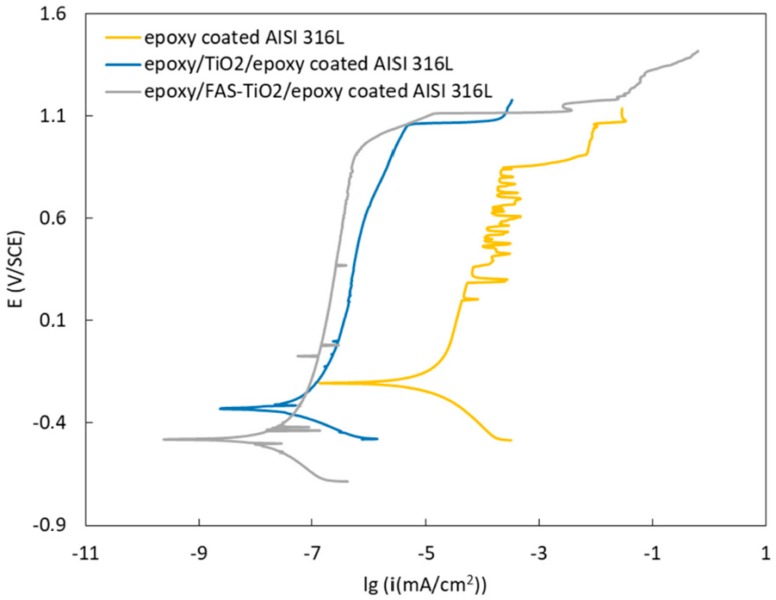
Potentiodynamic curves for samples in Hank’s solution.

We studied the potentiodynamic behaviour of the epoxy/FAS-TiO_2_/epoxy, epoxy/TiO_2_/epoxy and epoxy coatings on AISI 316L stainless steel in Hank’s solution (Figure 7). The quantitative results of the potentiodynamic measurements are presented in Table 2. We calculated the corrosion rate (*v_corr_*) and the corrosion current density (*i*_corr_) according to ASTM G102-89 [29]. The surface modification greatly influenced the polarization and the passivation behaviour of the substrate. The corrosion potential (*E*_corr_) for the epoxy coating was approximately −204 mV vs. SCE. After the Tafel range, the specimen displayed a wide passive region, followed by the breakdown potential (*E*_b_) at 800 mV vs. SCE. The corrosion rate was 0.23 µm/year. To achieve improved corrosion properties, we prepared coatings by the addition of the as-received and surface-modified TiO_2_ nanoparticles. The increased corrosion resistance of these samples was observed with a significantly wider range of passivation, which was shifted to lower corrosion-current densities (*i*_corr_). For the epoxy/TiO_2_/epoxy-coated AISI 316L sample, the *E*_corr_ was −329 mV vs. SCE and the *E*_b_ was shifted to 1000 mV vs. SCE. The corrosion rate was 0.8 nm/year. For the epoxy/FAS-TiO_2_/epoxy-coated AISI 316L sample, the *E*_corr_ was −479 mV vs. SCE and the *E*_b_ was 1000 mV vs. SCE. The corrosion rate was 0.4 nm/year. The results show the significantly improved corrosion resistance of the AISI 316L stainless steel after the addition of nanoparticles to the epoxy coating compared to the pure epoxy coating; this effect was especially noticeable for the hydrophobic epoxy/FAS-TiO_2_/epoxy coating. The key advantage is the presence of nanoparticles within the coating, which inhibits the permeation of aggressive ions and increases the corrosion performance of the substrate.

## 4. Conclusions

We studied the influence of surface morphology and wettability on the friction and wear resistance of hydrophilic pure epoxy, hydrophobic epoxy/FAS-TiO_2_/epoxy and hydrophilic as-received epoxy/TiO_2_/epoxy coatings. Experiments were performed in dry conditions, distilled water and in Hank’s solution. In the case of dry sliding, a lower coefficient of friction was achieved for both TiO_2_ reinforced coatings in comparison to the base epoxy coating due to the abrasive wear of the top epoxy layer and accumulation of the coating material around TiO_2_ nanoparticles forming larger load-carrying low-friction patches. In the water environment the same level of friction was shown for all three coatings, indicating that the friction level is defined by the water itself. In the Hank’s solution, however, we observed that the wettability and the implementation of the TiO_2_ nanoparticles regulate the friction level through the reactions between the solution and the elements of the coating revealed by abrasive wear. Friction was reduced for the hydrophilic epoxy/TiO_2_/epoxy coating, increased for the hydrophobic epoxy/FAS-TiO_2_/epoxy and had no effect on the pure epoxy coating. Finally, the results have shown that the reinforcement with the TiO_2_ nanoparticles provides better polymer coating resistance to mechanical and tribological loading provided by the formation of the patches of the coating material rich in Ti. The nanoparticle implementation within the coating also resulted in an enhanced corrosion resistance for the hydrophobic epoxy/FAS-TiO_2_/epoxy coating compared to the hydrophilic pure epoxy and the epoxy/TiO_2_/epoxy coatings.

## Figures and Tables

**Table 1:** Contact angles (θ^W^ and θ^Hank^) and average surface roughness (*S*_a_) of epoxy, epoxy/FAS-TiO_2_/epoxy and epoxy/TiO_2_/epoxy coatings.

Substrate	θ^W^ (^o^)	θ^Hank^ (^o^)	*S*_a_ (nm)
Epoxy	74.5 ± 1.6	77.7 ± 2.0	67.4 ± 6.0
Epoxy/FAS-TiO_2_/epoxy	121.4 ± 1.8	120.2 ± 2.1	702.8 ± 12.0
Epoxy/TiO_2_/epoxy	76.0 ± 1.0	73.8 ± 1.4	512.6 ± 10.0

**Table 2:** Electrochemical parameters determined from the potentiodynamic curves.

Material	*E* (*I* = 0) (mV)	*i*_corr_ (nA/cm^2^)	*v*_corr_ (nm/year)
Epoxy on AISI 316L	−204.4	20.000	226.5
Epoxy/TiO_2_/epoxy on AISI 316L	−329.2	0.069	0. 8
Epoxy/FAS-TiO_2_/epoxy on AISI 316L	−479.3	0.032	0. 4
